# Creating an algorithm to identify indices of sleep quantity and quality from a wearable armband in adults

**DOI:** 10.5935/1984-0063.20220052

**Published:** 2022

**Authors:** Laura Grau, Jaron Arbet, Danielle M Ostendorf, Jennifer M Blankenship, Shelby L Panter, Victoria A Catenacci, Edward L Melanson, Seth A Creasy

**Affiliations:** 1 University of Colorado Anschutz Medical Campus, Department of Biostatistics and Informatics - Aurora - CO - United States; 2 University of Colorado Anschutz Medical Campus, Division of Endocrinology, Metabolism, and Diabetes - Aurora - CO - United States; 3 University of Colorado Anschutz Medical Campus, Anschutz Health and Wellness Center - Aurora - CO - United States; 4 Eastern Colorado VA, Geriatric Research, Education, and Clinical Center - Aurora - CO - United States; 5 University of Colorado Anschutz Medical Campus, Division of Geriatrics - Aurora - CO - United States

**Keywords:** Validation Study, Actigraphy, Wearable Electronic Devices

## Abstract

**Objective:**

To develop an algorithm to quantify indices of sleep quantity and quality using the SenseWear armband (SWA) and to compare indices of sleep from this novel algorithm to standard wrist actigraphy (Actiwatch 2; AW2) under free-living conditions.

**Material and Methods:**

Thirty participants (47±10 years; 33.0±4.8kg/m^2^) wore the SWA and AW2 for seven consecutive days. Participants self-reported bedtime and waketime across these 7 days. Bedtime, sleep onset, sleep offset, waketime, total sleep time (TST), time in bed (TIB), sleep effciency (SE), sleep onset latency (SOL), wake after sleep onset (WASO), sleep fragmentations (SF), sleep regularity (calculated as SD of waketime), and mid-point of sleep were calculated using each device.

**Results:**

There was significant evidence for equivalence of means (or mean ranks) for bedtime, sleep onset, sleep offset, waketime, TST, TIB, SOL, WASO, and midpoint of sleep measured by the SWA and AW2 (*p*<0.05). There was insuffcient evidence for equivalence of means in SF (SW: 25±6 vs. AW2: 10±3 events; *p*=1.0), mean ranks in sleep regularity (SW: 58±33 vs. AW2: 68±40 min; *p*=0.11), and mean ranks in SE (SW: 84.7±5.1% vs. AW2: 86.3±5.5%; *p*=0.05). When comparing minute-by-minute sleep/wake status, the sensitivity and specificity of the SWA were 0.94 (95%CI: 0.93, 0.95) and 0.88 (95%CI: 0.85, 0.90), respectively, using AW2 as the criterion measure.

**Conclusion:**

The algorithm developed for the SWA produced relatively accurate and consistent measurements of sleep quantity, timing, and quality compared to the AW2 under free-living conditions. Thus, the SWA is a viable alternative to standard wrist actigraphy.

## INTRODUCTION

Polysomnography (PSG) is considered as the gold standard for measuring indices of sleep^1^. This technique, which is typically employed under laboratory conditions, includes continuous monitoring of brain waves, oxygen saturation, breathing patterns, skeletal muscle activity and body movements, and eye movement and requires a trained technician to perform sleep scoring^1^,^2^. Alternative, less invasive methods have been used to estimate sleep/wake behavior across populations under free-living conditions. The most common method to objectively assess free-living sleep/wake behavior is wrist actigraphy, which uses accelerometry to detect periods of movement and inactivity^3^,^4^. Wrist actigraphy is relatively inexpensive, unobtrusive, easy to analyze, and has been validated against PSG^5^,^6^. By defining sleep or wakefulness in minute-by-minute epochs, wrist actigraphy data can estimate indices of sleep duration and sleep quality. When compared to PSG, wrist actigraphy has been shown have high sensitivity (>90%; actigraphy = sleep when PSG = sleep), high accuracy (>90%; total proportion correct), but more variable and typically lower specificity (33%-96%) in adults^6^-^8^.

Consumer and research-grade wearable devices can also estimate parameters of sleep. Almost all wearable devices are equipped with accelerometers that can detect movement and posture, both of which can be used to discern sleep/wake behaviors. In addition, some devices have additional sensors (e.g., light-sensor, thermal sensors, electrocardiographs, gyroscopes, and photoplethysmographs) that capture other physiological parameters which can be applied to further understand sleep/wake behavior. Several of these wearable devices have been validated against PSG or wrist actigraphy for measuring indices of sleep quantity and quality^9^. While most devices identify time in bed and sleep duration, there are limited methods and resources available to capture other metrics of sleep (e.g., indices of sleep quality and timing) that may be related to health outcomes (e.g., obesity, type 2 diabetes, and depression).

The SenseWear Armband Mini (SWA; developed by BodyMedia Inc., Pittsburgh, PA) is a wearable activity monitor that has been commonly used in clinical research. The accuracy and validity of the SWA for measuring indices of sleep quality and quantity under free-living conditions is unknown. The objective of this analysis was to: 1) develop an algorithm to quantify indices of sleep quantity and quality from the SWA; 2) to compare these indices to the widely accepted method of wrist actigraphy (Actiwatch 2, AW2; Philips Respironics, Bend, OR); 3) and make the developed algorithm open-source and adaptable to other wearable devices that provide epoch-level data.

## MATERIAL AND METHODS

### Participants

This study leveraged data collected as part of a study followed individuals who completed an 18-month weight loss intervention at the University of Colorado - Anschutz Medical Campus (NCT01985568). The objective of the parent study was to evaluate the effects of two different behavioral weight loss interventions on change in body weight, body composition, and cardiorespiratory fitness. These findings have been published previously^10^. As part of an ancillary study, individuals who completed the weight loss intervention were invited for an assessment visit 3 years after the completion of the intervention. The present study utilized participants enrolled in this ancillary study. The Colorado Multiple Institutional Review Board approved all study procedures and participants provided informed consent prior to data collection. Eligibility criteria included adults aged 18-55 years with overweight and obesity (BMI≥27.0 to 42.0kg/m^2^) who were not physically active (self-reported <150min/wk of moderate intensity physical activity). Exclusion criteria included: significant cardiovascular metabolic and thyroid disease, cancer within the past 5 years, contraindications to exercise, previous weight loss surgery, eating disorder, medications affecting body weight, nicotine use, current or recent pregnancy.

### Assessment of sleep

Indices of sleep quantity and quality were assessed using wrist actigraphy (AW2) and the SWA. Participants were asked to wear each device for 24 hours (h) per day (d) for seven consecutive days. The AW2 was worn on the non-dominant wrist and the SWA was worn on the upper left arm. The SWA and AW2 were only removed during showering, bathing, and swimming. Valid days for both devices were defined as having at least 95% wear-time (1,361min/d). Because this analysis was focused specifically on sleep, days were classified as 12:00 to 11:59 the next day. The SWA and the AW2 epoch data were aligned, so only days with valid data for both devices were analyzed. For weekly summary analyses, participants needed to have valid data on ≥2 weekdays and ≥1 weekend day. Participants were also asked to keep a sleep/wake log by recording the time they went to bed (bedtime), the time they woke up (waketime), the time of naps if applicable, and if they removed either device during the observation period.

### Estimating sleep using AW2

The AW2 sleep data was scored using a standardized method^11^. Data were collected in minute-by-minute epochs and rest intervals (i.e., intervals from bedtime to waketime) were set using the following criteria: participant provided event markers at bedtime and waketime, self-reported sleep/wake log, defined activity/light thresholds, all of which were determined by manual inspection by trained study staff^11^. Data were scored as active (i.e., awake and not attempting to sleep), rest (i.e., awake but attempting to sleep), or sleep using manufacture provided software and a wake threshold of 40 counts (Actiware v. 6.0.9; Philips Respironics). The following indices of sleep were calculated: 1) bedtime - start of rest; 2) sleep onset - first time sleep is recorded; 3) sleep offset - period of wakefulness following a bout of sleep which is not followed by more sleep; 4) waketime - time out of bed in the morning; 5) total sleep time (TST) - sum of minutes classified as sleep between sleep onset and sleep offset; 6) time in bed (TIB) - sum of minutes between bedtime and waketime; 7) sleep efficiency (SE, %) - percent of time classified as sleep between sleep onset and sleep offset; 8) sleep onset latency (SOL) - minutes from bedtime until sleep onset; 9) wake after sleep onset (WASO) - sum of minutes scored as awake between sleep onset and sleep offset; 10) sleep fragmentations (SF) - sum of independent occasions scored as awake between sleep onset and offset; 11) sleep regularity - the standard deviation (SD) of waketime; and 12) midpoint of sleep calculated as the halfway point between sleep onset and sleep offset. Minute-by-minute, daily data, and weekly summary data were used in analyses. The AW2 has been shown to have accuracy ranging from 88-89%, sensitivity ranging from 91-97%, and specificity ranging from 39-66% when compared to PSG in adults^12^,^13^.

### Estimating sleep using SWA algorithm

**Figure 1:**
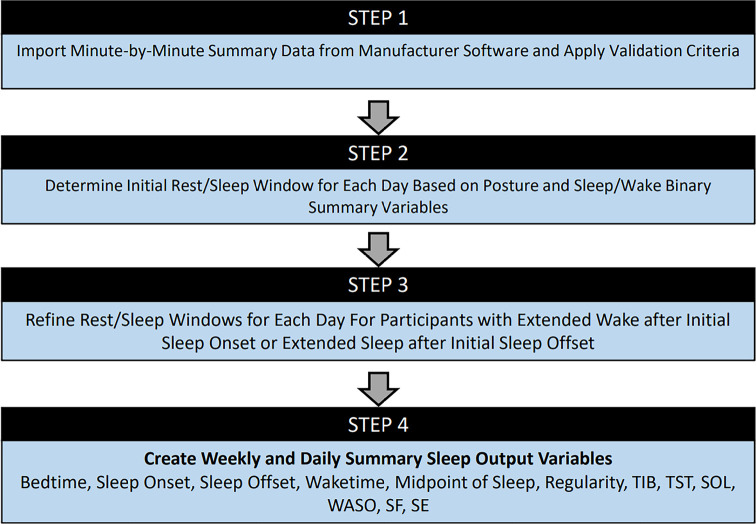
SAS macro algorithm criteria.

Notes: This figure demonstrates the flow of data using the created SAS Macro to score the minute-by-minute armband data. BT = Bedtime; S-On = Sleep onset; S-off = Sleep offset; TIB = Time in bed; TST = Total sleep time; SE = Sleep efficiency; SOL = Sleep onset latency; WASO = Wake after sleep onset; SF = Sleep fragmentations.

The SWA utilizes multiple sensors (accelerometer, skin temperature, heat flux, galvanic skin response) and a proprietary algorithm developed by the manufacturer to characterize body position, sleep/wake, and physical activity intensity data into minute-by-minute epochs. Summary data includes TST, but sleep timing and indices of sleep quality are not quantified from the manufacturer software. The goal of this study was to improve the accuracy and validity of the manufacturer sleep/wake algorithm by applying additional criteria to better characterize indices of sleep quantity and quality. Figure 1 illustrates an overview for how our SAS Macro code utilized the minute-by-minute data to create daily and weekly summaries of the sleep variables. The SAS Macro imported the following variables from the manufacturer minute-by-minute files: date-time, a binary indicator for lying down, and a binary indicator for sleep. During step 1, the Macro cleaned the data by requiring user-inputted validation criteria on number of minutes required for a valid day and valid number of weekdays and weekend days required for a valid week. Next, the Macro defined the potential nighttime sleep window based on user input: 19:00-11:59 the following day. During steps 2 and 3, the Macro searched the minute-by-minute data to define the sleep window (sleep onset to sleep offset), defined as the first time during the defined night where the participant was classified as lying down and asleep until the last time during the defined night where the participant was classified as lying down and asleep. If the initial sleep onset was followed by extended wake time (≥10 minutes) prior to 24:00 then the start of the sleep window was redefined to the first minute identified sleep prior to 24:00. If the participant was awake at 24:00 then the first minute identified as sleep after 24:00 was used as the start of the sleep window. The sleep window continued into the next day until the last minute of sleep, which was followed by ≥90 minutes of upright time. Once the sleep window was defined, the Macro determined time in bed (TIB) by searching for the first minute of lying down prior to sleep onset. This was defined as Bedtime. Next the Macro searched for the last minute of lying down following sleep offset. This was defined as waketime. The window from bedtime to waketime was defined as TIB. Midpoint of sleep, TST, WASO, SOL, SF, and SE were all determined using the same definitions as the AW2 above. The Macro utilized the sleep/wake binary variable from the manufacturer software and searched within each TIB or sleep window to determine these metrics of sleep quantity and quality. Criteria and rationale for these variables followed previously published procedures^14^. The SAS Macro is openly available at: https://github.com/graulaurak/sleep^15^. While the Macro and criteria within the Macro are designed to analyze specific input data, the macro is modifiable for other devices that provide epoch-level data.

### Statistical analyses

Statistical analyses were performed using SAS 9.4 (SAS Institute Inc., Cary, NC). The AW2 was classified as the standard criterion for assessing free-living sleep variables. Thus, our primary comparisons were indices of sleep as determined by the SWA compared to the AW2. Data are reported and were analyzed as person-days (i.e., each day for each subject counted separately) and weekly summaries (i.e., mean ± SD for each subject calculated across valid days). For weekly summaries, variables were compared between the two devices using two one-sided tests for paired samples or Wilcoxon Signed-Rank. These analyses were performed using NCSS 2021 Statistical Software v21.0.2 (NCSS. LLC; Kaysville, UT). Equivalence margins were specified *a priori* as 30 minutes for bedtime, sleep onset, sleep offset, waketime, midpoint of sleep, TIB, and TST; 10 minutes for regularity of sleep, SOL, and WASO; 5 events for SF; 3% for SE. We also compared bedtimes and waketimes from AW2 and SWA to those from the self-reported sleep logs using two one-sided tests with 30-minute margins.

Minute-by-minute data from the AW2 and the SWA were compared using multiple methods. The proportion of AW2 minutes correctly identified as sleep by the SWA (sensitivity) and the proportion of AW2 minutes correctly identified as awake by the SWA (specificity), as well as the corresponding positive and negative likelihood ratios were calculated using the epoch data. Sleep indices were compared using Bland-Altman plots, depicting the relationship between the mean SWA and AW2 variables and the difference (SWA - AW2). Within-subject correlation was accounted for in the calculation of the mean difference and limits of agreement in the Bland-Altman plots. Generalized estimating equation (GEE) models with working independence were used to quantify the bias in the Bland-Altman plots. Intra-class correlations (ICCs) were calculated for the sleep variables between SWA and the AW2^16^.

## RESULTS

**Figure 2:**
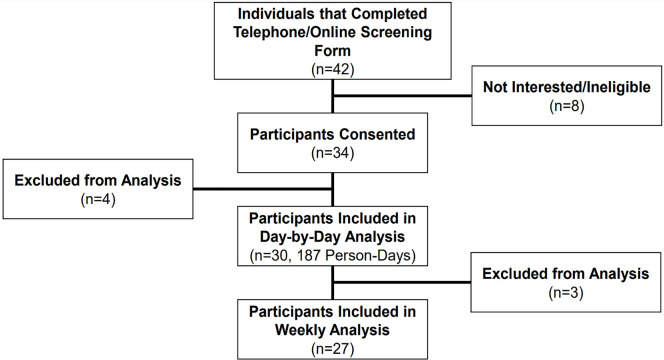
Consort diagram.

Thirty participants had ≥1 day of valid data for both the SWA and the AW2, for a total of 187 person-days (Figure 2). Four participants were excluded from all analyses due to insufficient wear time, device malfunction, or evidence of nighttime shift work. Twenty-seven participants had valid weekly data (defined as having >2 valid weekdays and >1 valid weekend day). Participants had an average of 6.3±1.7 days of valid data with both devices. Participant characteristics are in included in Table 1.

**Table 1:** Participant characteristics.

	n (%) or mean ± SD
**Sex**	
Female	24 (80%)
Male	6 (20%)
**Ethnicity**	
Not Hispanic/Latino	20 (67%)
Hispanic/Latino	10 (33%)
**Race**	
White	21 (70%)
Black	6 (20%)
Other	3 (10%)
**Age (years)**	46.7 ± 9.9
**BMI (kg/m^2^)**	33.0 ± 4.8
**Weight (kg)**	91.7 ± 17.8
**Self-reported health conditions**	
Cardiometabolic disorders	14 (47%)
Sleep and breathing disorders	6 (20%)
Mental health disorders	7 (23%)
Musculoskeletal pain	14 (47%)
**Valid days**	6.3 ± 1.7

There was significant evidence for equivalence of means (or mean ranks) for bedtime, sleep onset, sleep offset, waketime, TIB, TST, SOL, WASO, and midpoint of sleep (Table 2). There was insufficient evidence for equivalence of means in SF (SW: 25±6 vs. AW2: 10±3 events; *p*=1.0), mean ranks in sleep regularity (SW: 58±33 vs. AW2: 68±40 min; *p*=0.11), and mean ranks in SE (SW: 84.7±5.1% vs. AW2: 86.3±5.5%; *p*=0.05). There was significant evidence for equivalence of means between the bedtimes and waketimes calculated using the SWA or the AW2 compared to the self-reported times (*p*>0.05). Average self-reported bedtimes and waketimes were 22:44 ± 1:00 and 06:46 ± 1:02.

**Table 2:** Two one-sided tests for equivalence.

Sleep variable	AW2	SWA	p-value
**Bedtime (HH:MM)**	22:48 ± 01:01	22:41 ± 01:05	**<0.001**
**Sleep onset (HH:MM)**	23:03 ± 01:02	22:55 ± 01:04	<0.001^*^
**Sleep offset (HH:MM)**	06:35 ± 01:07	06:43 ± 01:16	<0.001^*^
**Waketime (HH:MM)**	06:47 ± 01:07	06:56 ± 01:18	<0.001^*^
**TIB (min/d)**	479.3 ± 60.1	485.4 ± 80.0	<0.001^*^
**TST (min/d)**	405.4 ± 51.1	418.2 ± 69.7	**0.02**
**SE (%)**	84.7 ± 5.1	86.3 ± 5.5	0.05^*^
**SOL (min/d)**	13.6 ± 13.5	14.0 ± 8.7	<0.001^*^
**WASO (min/d)**	48.8 ± 17.0	51.6 ± 29.8	0.02^*^
**SF (number of awakenings)**	24 ± 6	10 ± 3	1.00
**Midpoint of sleep (HH:MM)**	02:49 ± 00:58	02:49 ± 00:59	**<0.001**
**Sleep regularity (SD of waketime)**	58 ± 33	68 ± 40	0.11

Notes: Equivalence margins were specified a priori as 30 minutes for bedtime, sleep onset, sleep offset, waketime, midpoint of sleep, TIB, and TST; 10 minutes for regularity of sleep, SOL, and WASO; 5 for SF; 3% for SE. *Wilcoxon signed-rank tests.

**Figure 3:**
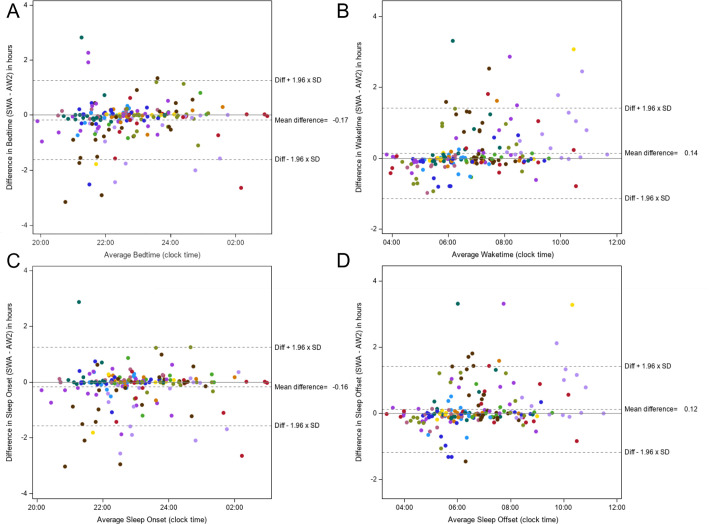
Differences in bedtime, waketime, sleep onset, and sleep offset.

Notes: A = Bland Altman illustrating differences in bedtime between the SWA and AW2; B = Bland Altman illustrating differences in sleep onset between the SWA and AW2; C = Bland Altman illustrating differences in sleep offset between the SWA and AW2; D = Bland Altman illustrating differences in waketime between the SWA and AW2; (n=187 person days); each color represents one subject’s data; x-axes are the average of the two device measurements, y-axes are the difference between SWA and AW2 (SWA - AW2).

**Figure 4:**
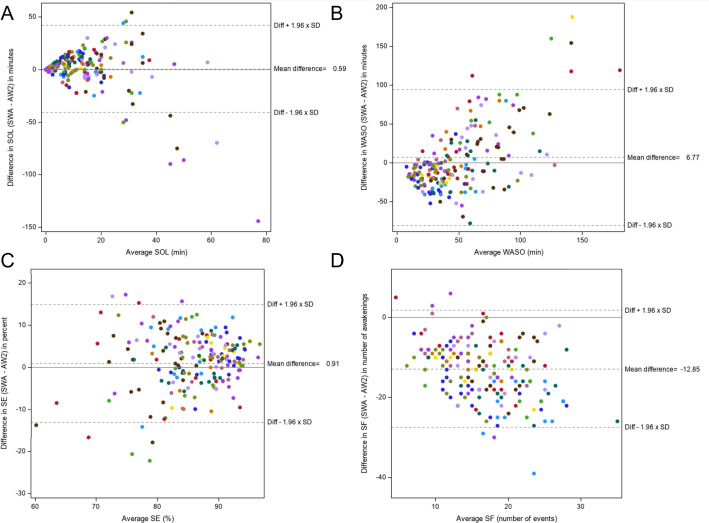
Differences in SOL, WASO, SE, and SF.

Notes: A = Bland Altman illustrating differences in sleep onset latency (SOL) between the SWA and AW2; B = Bland Altman illustrating differences in wake after sleep onset (WASO) between the SWA and AW2; C = Bland Altman illustrating differences in sleep efficiency (SE) between the SWA and AW2; D = Bland Altman illustrating differences in sleep fragmentations (SF) between the SWA and AW2; (n=187 person days); each color represents one subject’s data; x-axes are the average of the two device measurements, y-axes are the difference between SWA and AW2 (SWA - AW2).

Bland-Altman plots comparing person-day data on bedtime, sleep onset, sleep offset, and waketime from the SWA and AW2 are displayed in Figure 3. The SWA underestimated bedtime and sleep onset and overestimated sleep offset and waketime. There was a significant linear relationship between average waketime and the difference in waketime. For every 1 hour increase in waketime, the average difference increased by 0.14 (95%CI: 0.07, 0.20) hours (*p*<0.0001). There was also a significant linear relationship between average sleep offset and the difference in sleep offset. For every 1 hour increase in average sleep offset, the difference in sleep offset increased by 0.10 (95%CI: 0.04, 0.16) hours. There was no relationship between average bedtime and difference in bedtime (*p*=0.64) or between average sleep onset and difference in sleep onset (*p*=0.92). There were 20 (10.7%), 22 (11.8%), 24 (12.8%), 19 (10.2%) person-days with a >60-minute difference in bedtime, sleep onset, sleep offset, and waketime, respectively. Bland-Altman plots comparing person-day data on SOL, WASO, SE, and SF are shown in Figure 4. The SWA overestimated SOL and WASO and underestimated SF. There was a significant linear relationship between average WASO and the difference in WASO, as well as between the average SF and the difference in SF. For every one-minute increase in average WASO, there was, on average, a 0.82 (95%CI: 0.56, 1.09) minute increase in the difference (*p*<0.0001). For every one event increase in average SF, there was a 0.52 (95%CI: -0.76, -0.28) unit decrease in the difference of SF (*p*<0.0001). There was not a significant linear relationship between average SOL and the difference in SOL (*p*=0.09) or between average SE and the difference in SE (*p*=0.26). Sixty-five person-days (34.8%) had an absolute difference in WASO >30 minutes. 166 person-days (88.8%) had an absolute difference in SF of >5 awakenings.

In the minute-by-minute comparisons, across all participants, 91.1% of minutes at night had agreement in categorizing sleep and 93.7% had agreement in defining rest/lying down time between the SWA and AW2. The sensitivity across all minutes was 0.97 and the specificity was 0.85. The corresponding positive likelihood ratio was 6.51, and the corresponding negative likelihood ratio was 0.04. Sensitivity and specificity were also calculated across all nighttime minutes (19:00 until 11:59 the next day) for everyone to assess variation across individuals. Across individuals, mean sensitivity was 0.94 (95%CI: 0.93, 0.95) and mean specificity was 0.88 (95%CI: 0.85, 0.90). Intraclass correlations (ICC) for each sleep parameter are reported in Table 3. The ICC Shrout-Fleiss reliabilities for absolute agreement [ICC(2,1)] for bedtime, sleep onset, sleep offset, waketime, TIB, TST, WASO, SE, and SOL were good to excellent. The ICC for SF was poor. The ICC Shrout-Fleiss reliabilities [ICC(3,1)] for consistency for all variables were good to excellent.

**Table 3:** Intraclass Correlations (ICC) for inter-rater reliability.

Sleep variable	Absolute agreement ICC(2,1)	Consistency ICC(3,1)
**Bedtime**	0.78	0.78
**Sleep onset**	0.83	0.83
**Sleep offset**	0.87	0.87
**Waketime**	0.88	0.88
**TIB**	0.73	0.82
**TST**	0.76	0.79
**SE**	0.75	0.78
**SOL**	0.66	0.66
**WASO**	0.66	0.66
**SF**	0.13	0.75
**Midpoint of sleep**	0.65	0.65

TIB = Time in bed; TST = Total sleep time; SE = Sleep efficiency; SOL = Sleep onset latency; WASO = Wake after sleep onset; SF = Sleep fragmentations.

## DISCUSSION

There is a need to develop and refine tools to objectively assess parameters of sleep under free-living conditions. PSG is the gold standard measurement for sleep; however, it lacks utility for widespread use due to its high subject burden, need for technical expertise, and expense^1^. On the other end of the spectrum, subjective tools, such as questionnaires, can produce valid and reliable sleep data^17^; however, as with all self-report data, these instruments suffer from limitations and biases. Wearable devices are a good compromise, offering the ability to produce objective data on sleep quantity and quality, while being rather inexpensive and easy to use. In this study, we compared measures of sleep from the SWA to validated wrist actigraphy (AW2). The main finding from this study is that the algorithm, developed for the SWA, produced similar weekly average measures of sleep quantity, sleep timing, and most indices of sleep quality as compared to the AW2. Thus, the SWA may be a viable alternative for assessing aspects of sleep under free-living conditions. Findings from this study may provide serve as a guide for future studies seeking to identify the utility of wearable devices for measuring sleep in adult populations. Several large clinical trials that have previously utilized the SWA may be able to perform secondary analyses using the algorithm developed in this study. In addition, the algorithm and available code is adaptable and can calculate parameters of sleep quantity and quality from other research-grade (e.g., activPAL, Actiwatch, and Actigraph) and consumer-grade (e.g., Fitbit, Garmin, Apple, WHOOP, Polar, etc.) wearable devices that provide epoch-level data.

The SWA algorithm produced similar weekly estimates of bedtime, sleep onset, sleep offset, waketime, sleep regularity, and mid-point of sleep compared to AW2 and the self-reported sleep diaries. The SWA algorithm also produced a similar estimate of TST compared to AW2; this aligns with the performance of other wearable devices. Specifically, Mantua et al. (2016)^17^ found that the Actiwatch Spectrum and several consumer-grade wearable devices, including models from Fitbit, Misfit, Basis, and Withings, produced similar estimates of TST compared to PSG. A more recent study, found that the Fitbit Iconic and Oura smart ring produced accurate estimates of TST, total awake time, and sleep efficiency comparted to the a validated electroencephalography (EEG) device, whereas the other consumer devices (Apple Watch 3, Beddit Sleep Monitor, Fatigue Science Readiband, Garmin Vivosmart 4, Polar A370, and WHOOP strap 2.0) all had various degrees of bias and inaccuracy for these metrics^18^. Another recent study found that the Fatigue Science Readiband, Fitbit Alta HR, Garmin Fenix 5S, and Garmin VivoSmart 3 performed similarly to the AW2 and performed well at detecting sleep compared to PSG^13^. Because the SWA performed similarly to AW2, it is likely that it performs similarly to current consumer wearable devices for estimating TST.

Research and consumer devices have made significant efforts in the past few years to capture indices of sleep quality (sleep staging, SE, SOL, WASO, awakenings). We found that the SWA provided similar estimates of SOL and WASO, but significantly overestimated SE and number of awakenings per night compared to AW2. Chinoy et al. (2021)^13^ found that the Fatigue Science Readiband and Fitbit Alta HR produced relatively accurate estimates of SE compared to PSG, while the AW2, Garmin Fenix 5S and Garmin Vivosmart 3 overestimated SE. In that same study, most consumer devices produced significantly different estimates of SOL and WASO compared to PSG^13^. The magnitude of difference between these devices and PSG for SOL was rather small (<5 minutes) while the differences in WASO were much more variable (2.1-49.5min). Other studies have also found various levels of agreement between consumer devices and EEG or PSG. Stone et al. (2020)^18^ found that the Oura and Fitbit produced similar estimates of SE compared to EEG but the Garmin and WHOOP were less accurate. There are several potential reasons for why consumer devices have produced more variable results in terms of these sleep quality metrics. These possibilities could be device specific, such as differences in sensor inputs, device hardware, device software, and the sensitivity of algorithms that determine sleep quality metrics. In addition, differences in the populations studied (age, weight, race/ethnicity, sleep disorders) and differences in wear protocols (number of days, free-living vs. in-laboratory) may influence the accuracy of these devices. Overall, the SWA suffers from similar limitations as other consumer and research grade devices when trying to assess sleep quality. In addition, several consumer-grade devices claim to capture sleep stages (e.g., light vs. deep sleep). The SWA does not have the capabilities of sleep staging; thus, it was not a focus of this analysis. Further, validation studies have questioned the accuracy and validity of sleep staging estimates from most devices.

Wearable devices that rely on movement to detect sleep/wake have inherent limitations detecting true wake, especially motionless wake. Thus, wearable devices typically have lower specificities ranging from 27-77% when compared to PSG^5^,^6^,^8^,^19^,^20^. The specificity of the SWA was 85% when compared to all minutes from the AW2. These data suggest that the SWA likely suffers from the same limitations as other wearable devices and would be unable to accurately detect true wake. In fact, the SWA algorithm was even less sensitive at detecting wakefulness across the night compared to AW2 as evidenced by the lower SF and higher SE observed in the SWA. Without a PSG measurement in this study, it is difficult to discern which device was more accurate; however, a previous study found that the Actiwatch Spectrum provided similar SE estimates compared to PSG^21^. Additional sensors or more complex algorithms may be necessary to improve the specificity of the SWA and other wearable devices. For example, wearable devices with red and infrared photoplethysmographs offer the best estimates of heart rate and heart rate variability during sleep which may help to provide more accurate estimates of sleep/wake and sleep staging^22^.

When comparing individual-level data from the SWA to the AW2, the SWA algorithm was less accurate and more variable. Other studies have also found that wearable devices have limited capability to accurately capture individual level data^9^. The poor daily estimates in this study were driven by a few specific participants. It is possible that the characteristics of sleep (i.e., body movements, posture, body positioning, etc.) in these participants made it difficult to classify sleep/wake behavior using the SWA. There were also differences between the hardware in the devices, how the devices are worn, and how the data are processed that may have caused the incongruity between measures of sleep from each device. First, the SWA and the AW2 may have been worn on opposite arms and have different wear locations (wrist vs. upper arm). The SWA utilized multiple inputs (movement, heat flux, skin temperature, and galvanic skin response) to determine sleep vs. wake while the AW2 uses movement and light exposure to determine sleep vs. wake. Additionally, these devices utilize different processing algorithms when deciphering between wake and sleep. Future studies should investigate the root cause of these discrepancies between devices.

The algorithm that was created to process SWA data and create the indices of sleep is freely available and adaptable for use with other wearable devices. In the current form the algorithm utilizes inputs based on user-specified validation criteria (number of hours per day, number days per week, etc.), postural changes, and a binary sleep/wake variable determined from the manufacturer’s proprietary software which utilized information from the accelerometer, galvanic skin response, skin temperature, and heat flux. Future studies could adapt this algorithm to other wearable devices that provide epoch level data. Algorithm inputs could be modified to include measurements of raw acceleration, skin temperature, heart rate, blood pressure, gyroscope, light exposure, etc. Certain inputs may be more or less important based on the metric of sleep that is being estimated. This algorithm may have utility for both research-grade and consumer-grade devices.

There are several limitations in this study. First, this study used wrist actigraphy as the criterion measure of free-living sleep measures; PSG is considered the gold-standard, but it was not feasible for this study. This study used a small, convenient sample of participants enrolling in a follow-up study to a behavioral weight loss intervention. Although this subject population was diverse, it may not be representative of all adult populations, as the subjects were primarily female with overweight or obesity. Importantly, the enrolled participants did not have diagnosed sleep problems, so this algorithm may not be accurate in populations with known sleep disorders. The algorithm used to analyze the SWA data utilized data inputs, which included factors related to time of day, posture, and a binary sleep/wake variable. Not all wearable devices provide such information; however, the algorithm can be modified to include a variety of inputs and may be adapted to other wearable devices. Because the algorithm utilized time of day to determine nighttime sleep, the current code cannot be used to discern daytime sleep and napping behavior. In addition, the SWA and current algorithm likely do not improve detection of true wake, a common limitation of wearable devices. Finally, we recognize that the SWA is no longer commercially available; however, this analysis may be useful for analyses of existing datasets with 24h SWA data and inform clinical and research settings using the SWA. Additionally, the findings from this study may be useful for other studies investigating the utility of wearable devices for measuring indices of sleep under free-living conditions.

In summary, the SWA algorithm produced weekly summary measures of bedtime, sleep onset, sleep offset, waketime, midpoint of sleep, TST, TIB, SOL, and WASO that were consistent with wrist actigraphy (AW2). While the ICCs calculated in the day-to-day comparisons showed that there is generally good absolute agreement and consistency between the devices, there was significant bias in SE, SOL, and SF. The differences in these measures are likely due to inconsistencies in minute-by-minute data due to differences in device sensors as well as varying levels of sensitivity thresholds for sleep/wake. While we did not assess sleep using other wearable devices, it seems likely that SWA suffers many of the same limitations when estimating sleep as compared to other consumer devices. Taken together, the SWA is suitable for measuring indices of sleep quantity, timing, and quantity in adults without sleep disorders. Our open-source algorithm may be applied to previous studies that have collected 24h SWA data and to other devices, both research and consumer grade, to calculate parameters of sleep quantity and quality.
